# Estimates of Uterine Rupture Bad Outcomes Using Propensity Score and Determinants of Uterine Rupture in Mizan-Tepi University Teaching Hospital: Case Control Study

**DOI:** 10.1155/2017/6517015

**Published:** 2017-07-09

**Authors:** Tegene Legese Dadi, Teklemariam Ergat Yarinbab

**Affiliations:** Department of Public Health, College of Health Sciences, Mizan-Tepi University, Mizan-Aman, SNNPR, Ethiopia

## Abstract

**Background:**

Uterine rupture is a tear in the wall of uterus which carries grave risks to the mother as well as her baby.

**Objectives:**

To estimate uterine rupture bad outcomes using propensity score and its determinants in Mizan-Tepi University teaching hospital.

**Methods:**

A case control study on 363 participants, 121 cases and 242 controls, was conducted. Data was analyzed by STATA 14. Propensity score matching analysis was used to see causes. Level of significance of *p* value is ≤0.05.

**Results:**

Females who reside in rural areas (AOR = 3.996; 95% CI: 2.011, 7.940) are at higher risk of acquiring uterine rupture. Females who had ANC follow-up (AOR = 0.315; 95% CI: 0.164, 0.606) and preterm gestational age (AOR = 0.135; 95% CI: 0.025, 0.725) are at lower risk of developing uterine rupture. Propensity score matching analysis shows that, from 100 participants who had uterine rupture, 88.4 females lost their fetus (*β* = 0.884; 95% CI: 0.827, 0.942). From 100 females who develop uterine rupture, 9.1 died (*β* = 0.091; 95% CI: 0.040, 0.142). From 100 females who develop uterine rupture, 97.5 developed additional obstetric complication (*β* = 0.975; 95% CI: 0.947, 1.000).

**Conclusion:**

Residence, ANC follow-up, and gestational age are significant determinants of uterine rupture. Fetal loss, maternal death, and obstetric complications are significant bad outcomes of uterine rupture.

## 1. Introduction

Uterine rupture is a rip or a tear in the wall of the uterus due to pregnancy or delivery. It is one of rare obstetric complications. Systematic review done by WHO shows that the prevalence is lower in developed countries than in the less or least developed countries. It is 0.006% in developed country but may reach up to 25% for women with obstructed labor in a least developed country as per the previous review [1].

The prevalence of uterine rupture may vary in different health institutions in different countries. In Adigrat Zonal Hospital (Ethiopia), in Kandang Kerbau Hospital (Singapore), in Yemen, and in Niger Delta Hospital (Nigeria), its prevalence is 0.9% (1 : 110), 0.02% (1 : 6331), 0.63%, and 0.04% (1 : 2572), respectively [2–5].

The worry for uterine rupture is due to its dreadful outcomes. It carries grave risks to the mother and her baby. Even if women survive, the future reproductive potential is brought down or turned a loss forever. Majority of ruptured uteruses are traumatic [6].

As the prevalence is higher, its consequences are also more severe in less and least developed nations. Maternal death, fetal demise, still birth, and intrauterine fetal death are severe consequences. In Mbarara University of Science and Technology teaching hospital, case fatality rate of uterine rupture was 12% [7]. In Kandang Kerbau Hospital, Singapore, the overall incidence of fetal loss was 7.4% [3]. In Lahore General Hospital, the incidence of uterine rupture was 0.29%, among which 7 mothers lost their uterus and there were 10 (71.4%) intrauterine deaths [8]. For maternal outcome in Sana'a city, Yemen, almost half of patients (45.7%) had a repair of the rupture and 20% of women lost their future productivity. There was 54.3% of fetal death [4].

Obstructed labor due to cephalopelvic disproportion and malpresentation, previous cesarean section, oxytocin infusion, and grand multiparity are the major direct factors for uterine rupture. In Sana'a city, Yemen, obstructed labor was found in 83.3% and contracted pelvis was found in 19.4% of cases. Seven cases (19.5%) of uterine rupture in this study had a history of caesarean section. Fetal weight is a risk only in one case (fetal weight > 3500 g) [4].

The antecedent predisposing factors for uterine rupture in different hospitals were previous caesarean section, attending <4 antenatal visits, parity ≥5, and no formal education [4, 7, 8]. In addition, induction of labor, high birthweight, postterm births, maternal age ≥ 35 years, and short maternal stature were also associated with increased risk of uterine rupture [9].

## 2. Significance for Public Health

The purpose of this study was to estimate antecedent determinant risk factors of uterine rupture and to estimate the outcomes of uterine rupture using propensity score matching analysis. It is also useful to extrapolate the outcomes of uterine rupture for wider population using the above mentioned analysis. So this study is helpful to determine what factors affect uterine rupture and to determine the amount of loss due to uterine rupture using statistical quantification. Thus the result contributes to designing context-specific intervention mechanisms.

## 3. Objective

### 3.1. General Objective

The general objective is to estimate bad outcomes of uterine rupture using propensity score matching analysis and to assess determinants of uterine rupture in Mizan-Tepi University teaching hospital (MTUTH) from September 2011 to August 2016 G.C.

### 3.2. Specific Objectives

The specific objectives are to determine determinants of uterine rupture in MTUTH from September 2011 to August 2016 G.C and to determine bad outcomes of uterine rupture using propensity score matching analysis in MTUTH from September 2011 to August 2016 G.C.

## 4. Methods

### 4.1. Study Setting

The study was conducted in the Department of Gynecology and Obstetrics in MTUTH which is located in Bench Maji zone, Southern Nations, Nationalities, and Peoples' Region (SNNPR), Ethiopia. It is 583 kilometers off from Addis Ababa in southwest direction. This hospital was founded in 1987 G.C. It serves more than 1 million people. Now the hospital has 17 medical doctors, 4 specialists, 79 nurses, 75 support staff members, and 150 administrative staff members. The weather condition is tropical rain forest and the topographic area is full of ups and downs.

#### 4.1.1. Study Design

Institution-based case control study was conducted.

#### 4.1.2. Source Population

The source population included all mothers who delivered at MTUTH.

#### 4.1.3. Study Population

Cases included charts of mothers who are diagnosed with uterine rupture due to pregnancy or delivery. Controls included charts of mothers who delivered by spontaneous vaginal delivery.

#### 4.1.4. Case Definition and Selection

Uterine rupture was defined as tearing of the uterine wall either partially or completely during pregnancy and labor, either diagnosed clinically or later confirmed at laparotomy. The cases were retrospectively collected from registers of Department of Gynecology and Obstetrics as well as from the patients' case files at the hospital's medical records office.

#### 4.1.5. Selection of Controls

Controls were women who had spontaneous vaginal delivery (SVD). For every case nearby two women who had SVD were used as controls.

#### 4.1.6. Inclusion and Exclusion Criteria

All women diagnosed with uterine rupture and selected SVD mothers were included. Those who had ruptured uterus and were managed at the peripheral hospitals and admitted to the MTU teaching hospital for the management of the complication are excluded.

### 4.2. Study Variables

#### 4.2.1. Dependent (Outcome) Variables

The study included uterine rupture and maternal and fetal outcomes of uterine rupture as dependent variables.

#### 4.2.2. Independent (Explanatory) Variables

The study included age of the mother, parity, residence (urban/rural), ANC, gestational age, preoperative hemoglobin, and sex of neonate as independent variables.

#### 4.2.3. Grand Multiparity

It describes a mother who gave birth more than five times in her life.

### 4.3. Data Collection

Data were collected by checklist adapted from maternal death surveillance technical guideline of Ethiopia [10]. To confirm whether the checklist is comprehensive it is pretested with patients' record files. The checklist contains the following categories of variables: sociodemographic information, obstetric and delivery history, the outcomes of pregnancy, and presence of obstetric complications, and other important variables are included. Data collection is performed by three middle-level midwives. The data collection is monitored and supervised by one public health officer and the principal investigator supervised the overall data collection process. To collect expected quality, data collectors and supervisor were trained for two days until they became well conversant with the instrument. The training includes the contents of the tool, ethical considerations, and way of data mining from the chart. After data collection, each filled checklist was given a unique code by the principal investigator.

### 4.4. Data Management and Analysis

The data was entered into Epi Info version 3.5.1. For analysis, the data is exported to STATA 14 (StataCorp, College Station, TX, USA). Any identified error was corrected with the revision of the original data using the code numbers.

Frequencies were used to check for entry errors, missed values, and outliers. Bivariate binary logistic regression was used to determine the association between different factors and the outcome variable. Variables that have *p* value less than or equal to 0.25 were included in the multivariate binary logistic regression to identify determinant factors of uterine rupture. The precision of the study was determined on 95% confidence interval (CI). The level of significance was taken as *p* value ≤ 0.05. The adjusted odds ratio with the 95% CI was reported.

Propensity score matching analysis was applied to find out the bad consequences of uterine rupture. It controls confounders by matching in exposed and nonexposed groups by calculating propensity score of variables. The cause is unrelated to confounders if participants have the same propensity score. Therefore, the cases and controls tend to have similar distributions of measured confounders other than cause, something that we would also achieve using randomization. Causal inference of uterine rupture on bad outcomes is determined at the level of significance of *p* value ≤ 0.05. The coefficient (*β*) is reported and interpreted in 95% confidence interval level of precision. Graphical presentation, such as tables, was used to present the result findings of the study.

### 4.5. Ethical Consideration

Permission was obtained from Mizan-Tepi University research and community service directorates and College of Health Sciences. Official letter was taken from MTUTH. The procedure and purposes of the study were explained to the hospital manager and to the hospital medical director. MTUTH gave permission to conduct the study. The patients' names were not included in the checklist. After finishing the data collection, the patients' documents were returned to card room.

## 5. Results

The overall incidence of uterine rupture is 1.24% (121 uterine rupture cases and 9789 total deliveries in the study period). The study was performed in 121 cases of uterine rupture and 242 controls of spontaneous vaginal delivery. The data is collected from total of 363 clients' charts. Marital status, monthly income, ethnicity, and religion are missing from the charts, so they are excluded from analysis.

Females who had favorable age groups for pregnancy (20–34) account for more than three-fourths of our samples. From females who had uterine rupture, 78.5% are in the age group of 20−34. Lowest number of uterine rupture cases is found in the age group of <20 years. Most of the clients come from semiurban areas; however, most of females with uterine rupture come from rural areas. From 363 females, 225 females are multiparous females. About two-thirds of females with uterine rupture had two to five children. More than 60% of females had ANC follow-up, but 60% of females who had uterine rupture do not have an ANC follow-up (Table 1).

More than three-fourths of uterine rupture cases occur during term gestational age. 85% of uterine rupture cases primarily have prolonged labor (labor length more than 24 hours). More than 85% of females who had uterine rupture are referred from other health institutions. More than 65% of females (245 out of 362) do not have hemorrhage; however, in contrast, more than 95% of females who had uterine rupture had hemorrhage. 80% of females had hemoglobin more than 7 mg/dL, but more than half of females who had uterine rupture have preoperative hemoglobin less than 7 mg/dL (Table 1).

All uterine rupture cases are seen by specialists. Three-fourths of fetal weight cases are in normal range. 92% of females who had uterine rupture had any types of risk factors (Table 1).

### 5.1. Determinant Factors of Uterine Rupture

In bivariate binary logistic regression, age groups of <20 and 20–34 years are protective age groups relative to reference group (age group > 34 years). Females in the age group of <20 years are 88% (COR = 0.12; 95% CI: 0.040, 0.370) and those in the age group of 20–34 years are 50.6% (COR = 0.494; 95% CI: 0.255, 0.957) less affected by uterine rupture relative to reference group. Females who came from rural areas are 3 times at higher risk of acquiring uterine rupture (COR = 3.168; 95% CI: 1.975, 5.082). Grand multipara females are at higher risk of uterine rupture. Nulliparous females have 90.1% (COR = 0.099; 95% CI: 0.042, 0.231) and multiparous females have 61.8% (COR = 0.382; 95% CI: 0.199, 0.736) less probability to develop uterine rupture relative to grand multipara females (Table 2).

Females who had ANC follow-up are at lower risk of developing uterine rupture by 78.3% (COR = 0.217; 95% CI: 0.136, 0.346). Females with postterm pregnancy are at higher risk of developing uterine rupture. Females with preterm and term gestational age pregnancies are at lower risk of developing uterine rupture by 88.6% (COR = 0.114; 95% CI: 0.034, 0.382) and 76.1% (COR = 0.239; 95% CI: 0.111, 0.516), respectively, as compared to females with postterm pregnancy. Even though the precision is low, females with preoperative hemoglobin level < 7 mg/dL are at higher risk of developing uterine rupture (COR = 32.842; 95% CI: 14.906, 72.360) (Table 2).

In multivariable binary logistic regression, ANC follow-up, residence, gestational age, and preoperative hemoglobin are significant predictors of uterine rupture. Females who reside in rural areas are 3.996 times at higher risk of acquiring uterine rupture (AOR = 3.996; 95% CI: 2.011, 7.940). Females who had ANC follow-up are at lower risk of developing uterine rupture, which is reduced by 68.5% (AOR = 0.315; 95% CI: 0.164, 0.606). Females with preterm gestational age pregnancy are at lower risk of developing uterine rupture, which is reduced by 86.5% (AOR = 0.135; 95% CI: 0.025, 0.725). Females with preoperative hemoglobin level < 7 mg/dL are at higher risk of developing uterine rupture (AOR = 62.517; 95% CI: 22.181, 176.204) but the confidence interval is wide which shows that the sample size is small to consider preoperative hemoglobin level < 7 mg/dL as determinant factor (Table 2).

### 5.2. Bad Outcomes of Uterine Rupture

Uterine rupture has deadly contributions to maternal and fetal loss. There are a total of 11 maternal deaths out of 363 females. Around 107 females lost their fetus. All of them are from uterine rupture cases. Around 7% of females have postoperative hemoglobin level less than <7 mg/dL and more than 30% of them have obstetric complications. More than 95% of females with uterine rupture have additional obstetric complications (Table 3).

In propensity score matching analysis, fetal loss, maternal death, and additional obstetric complications are significant bad outcomes of uterine rupture. In the participants in our study, 88.4 females lost their fetus per 100 females with uterine rupture due to uterine rupture (*β* = 0.884; 95% CI: 0.827, 0.942). 9.1 females per 100 females with uterine rupture died due to uterine rupture (*β* = 0.091; 95% CI: 0.040, 0.142). Additional obstetric complications were found in 97.5 per 100 females with uterine rupture (*β* = 0.975; 95% CI: 0.947, 1.000) (Table 4).

Bad outcomes of uterine rupture estimation by propensity score matching analysis for the population revealed that fetal loss, maternal death, and obstetric complications are still significant bad outcomes of uterine rupture. An increase of 1000 females who had uterine rupture in the community increases fetal loss by 812 (*β* = 0.812; 95% CI: 0.721, 0.904). An increase of 1000 females who had uterine rupture in the community increases maternal death by 35 (*β* = 0.035; 95% CI: 0.009, 0.610). An increase of 1000 females who had uterine rupture in the community increases additional obstetric complication by 992 (*β* = 0.992; 95% CI: 0.982, 1.000) (Table 5).

## 6. Discussion

### 6.1. Determinants of Uterine Rupture

Obstructed labor due to cephalopelvic disproportion and malpresentation, previous cesarean section, oxytocin infusion, and grand multiparity are the major direct factors for uterine rupture. The highest number of uterine rupture (78.5%) cases is found in the age group of 20−34 years and the lowest number of uterine rupture cases is found in the age group of <20 years. Even though age <20 years is significant in bivariate logistic regression, it is not significant predictor in multivariable logistic regression. Females in the age group of <20 years are 88% (COR = 0.12; 95% CI: 0.040, 0.370) less affected by uterine rupture relative to age group of >34 years. Similarly, in Uganda, teenagers were associated with less risk of developing ruptured uterus compared to those aged 20 to 29 years (OR: 0.1; 95%: 0.0–0.4) [11]. In Sweden, which is a developed country, high maternal age (>35 years) is 1.78 times more likely exposed for uterine rupture than age in the twenties (AOR = 1.78; 95% CI: 1.21–2.62) [9]. This might be due to the fact that mothers in this age group have experienced many pregnancies and deliveries. For instance, in this study, two-thirds of participants are grand multiparity females. This shows that pregnancy in age more than 34 or 35 years poses risk of uterine rupture.

Females who reside in rural areas are 3.996 times at higher risk of acquiring uterine rupture (AOR = 3.996; 95% CI: 2.011, 7.940) than females who reside in semiurban areas. Rural females in developing country are vulnerable to experience uterine due to inaccessibility of health facility, low level of literacy level, and other socioeconomic factors. For instance, in this study, only one-third of females come from rural area but more than half of them do not have ANC follow-up. Besides this there is a delay to visit health institutions in rural areas because of poor road and low literacy level.

Females who had ANC follow-up are at lower risk of developing uterine rupture, which is reduced by 68.5% (AOR = 0.315; 95% CI: 0.164, 0.606), than their counterparts. Similarly, in Uganda, females who do not have antenatal care are 4.7 times more likely to be exposed to uterine rupture (OR: 4.7; 95% CI: 1.6−13.7). In India, 70% of uterine rupture cases were unbooked cases for ANC [11, 12]. ANC is taken as the entry point for other maternal health care services. ANC service has a great contribution to identifying factors of exposure to uterine rupture. Thus females who had ANC follow-up are at lower risk of developing uterine rupture.

Being in the preterm gestational age is also one of the protective factors with AOR of 0.135 and 95% CI of 0.025–0.725 in contrast to postterm gestational age. In Sweden, the risk of uterine rupture was also increased in women with postterm (>42 weeks) compared with term (37–41 weeks) pregnancies [9]. If there is no spontaneous onset of labor in postterm pregnancies, the fate of pregnancy might be induction or operative delivery. Risk of uterine rupture doubled or tripled in induction delivery. Thus females with postterm pregnancies might be at higher risk of acquiring uterine rupture.

### 6.2. Estimates of Bad Outcomes of Uterine Rupture

The worry for uterine rupture is due to its dreadful outcomes. It carries terrible outcome to the mother and her baby. Even if women survive, the future reproductive potential is reduced or lost forever [6]. In this finding, more than 9% (11 out of 121) of females with uterine rupture are dead. But lesser percentages of maternal mortality were documented in Maputo and India, 7.3% and 2.76%, respectively [12, 13]. This is due to the fact that one-third of females come from rural area and half of them do not have ANC follow-up. The up and down topographic setup may hinder referral by increasing time on transportation. Terrible outcome of uterine rupture is also confirmed by propensity score matching analysis as described above in Results (Table 4). The fatality of uterine rupture is demonstrated by the fact that 9.1 out of 100 females who develop uterine rupture died due to uterine rupture (*β* = 0.091; 95% CI: 0.040, 0.142). But when it is extrapolated to community using the previous analysis its fatality is reduced to 3.5 (*β* = 0.035; 95% CI: 0.009, 0.610) as described in Table 5.

More than 85% of females with uterine rupture lost their fetus, a percentage that is lower than finding from India, 94.07%, but it is higher than finding from Lahore General Hospital, 71.4% [8, 12]. This difference might be due to setup difference and variation in included sample. In Ethiopia, one of the big headaches is the very high percentage of home delivery and low percentage of follow-up during pregnancy which may result in high percentage of loss of fetus in this finding. Study from Lahore General Hospital includes small number of samples, which may affect the precision of the study. The propensity score matching analysis confirmed its terrible outcomes on loss of fetus. From participants, 88.4 females per 100 females with uterine rupture lost their fetus due to uterine rupture (*β* = 0.884; 95% CI: 0.827, 0.942) but when it is extrapolated to the community its effect is reduced to 81.2 (*β* = 0.812; 95% CI: 0.721, 0.904).

The other dreadful contribution of uterine rupture is that it exposes the women to additional obstetric complications. More than 95% of females with uterine rupture have additional obstetric complications. From 100 females caught by uterine rupture, 97.5 developed additional obstetric complications (*β* = 0.975; 95% CI: 0.947, 1.000); however, when it is extrapolated to community, the percentage increased to 99.2 (*β* = 0.992; 95% CI: 0.982, 1.000).

These estimations imply that uterine rupture is the major concern particularly in developing countries. Its contribution to the fetal loss is horrible. For most of the terrible outcomes, strong follow-up of maternal and child health services during pregnancy and delivery is crucial to halt the predisposing factors for uterine rupture.

## 7. Conclusion

Residence, ANC follow-up, and gestational age are significant determinants and factors of uterine rupture. Fetal loss, maternal death, and obstetric complications are significant bad outcomes of uterine rupture. Residence and ANC follow-up are modifiable risk factors. Creating new way of referral system, where the area is difficult for the normal referral system, or increasing access of health services in the nearby area for difficult topographic areas is important to reduce the burden of uterine rupture.

As recommendation for future study, use of oxytocin and previous cesarean section with duration of hospitalization before uterine rupture should be studied.

## Figures and Tables

**Table 1:** Frequency distributions of sociodemographic and obstetric factors, MTUTH, 2016.

Variables	Category	SVD	Uterine rupture	Total
Age	<20 yrs	39 (88.6%)	5 (11.4%)	44 (100%)
		16.1%	4.1%	12.1%
	20–34 yrs	183 (65.8%)	95 (34.2%)	278 (100%)
		75.6%	78.5%	76.6%
	>34 yrs	20 (48.8%)	21 (51.2%)	41 (100%)
		8.3%	17.4%	11.3%

Residence	Urban	27 (81.8%)	6 (18.2%)	33 (100%)
		11.2%	5.0%	9.1%
	Rural	61 (48.8%)	64 (51.2%)	125 (100%)
		25.2%	52.9%	34.4%
	Semiurban	154 (75.1%)	51 (24.9%)	205 (100%)
		63.6%	42.1%	56.5%

Parity	Nullipara	81 (87.1%)	12 (12.9%)	93 (100%)
		33.5%	9.9%	25.6%
	Multipara	143 (63.6%)	82 (36.4%)	225 (100%)
		59.1%	67.8%	62.0%
	Grand multiparity	18 (40.0%)	27 (60.0%)	45 (100%)
		7.4%	22.3%	12.4%

ANC	No	60 (45.1%)	73 (54.9%)	133 (100%)
		24.8%	60.3%	36.6%
	Yes	182 (79.1%)	48 (20.9%)	230 (100%)
		75.2%	39.7%	63.4%

Gestational age	Preterm	23 (82.1%)	5 (17.9%)	28 (100%)
		9.5%	4.1%	7.7%
	Term	208 (68.6%)	95 (31.4%)	303 (100%)
		86.0%	78.5%	83.5%
	Postterm	11 (34.4%)	21 (65.6%)	32 (100%)
		4.5%	17.4%	8.8%

Duration of labor	<24 hrs	242 (93.1%)	18 (6.9%)	260 (100%)
		100.0%	14.9%	71.6%
	>24 hrs	0 (0.0%)	103 (100%)	103 (100%)
		0.0%	85.1%	28.4%

Referral	No	242 (93.4%)	17 (6.6%)	259 (100%)
		100.0%	14.0%	71.3%
	Yes	0 (0.0%)	104 (100%)	104 (100%)
		0.0%	86.0%	28.7%

Hemorrhage	No	242 (98.8%)	3 (1.2%)	245 (100%)
		100.0%	2.5%	67.5%
	Yes	0 (0.0%)	118 (100%)	118 (100%)
		0.0%	97.5%	32.5%

Preoperative hemoglobin	>7 mg/dL	234 (80.4%)	57 (19.6%)	291 (100%)
		96.7%	47.1%	80.2%
	<7 mg/dL	8 (11.1%)	64 (88.9%)	72 (100%)
		3.3%	52.9%	19.8%

Birth attendant	Gynecologists	0 (0.0%)	121 (100%)	121 (100%)
		0.0%	100.0%	33.3%
	Midwives	232 (100%)	0 (0.0%)	232 (100%)
		95.9%	0.0%	63.9%
	Others	10 (100%)	0 (0.0%)	10 (100%)
		4.1%	0.0%	2.8%

Fetal weight	<2.5 kg	2 (100%)	0 (0.0%)	2 (100%)
		0.8%	0.0%	0.6%
	2.5–4 kg	239 (86.0%)	39 (14.0%)	278 (100%)
		98.8%	32.2%	76.6%
	>4 kg	0 (0.0%)	2 (100%)	2 (100%)
		0.0%	1.7%	0.6%
	Undocumented	1 (1.2%)	80 (98.8%)	81 (100%)
		0.4%	66.1%	22.3%

Sex of neonate	Female	124 (70.1%)	53 (29.9%)	177 (100%)
		51.2%	43.8%	48.8%
	Male	118 (63.4%)	68 (36.6%)	186 (100%)
		48.8%	56.2%	51.2%

Risk factors composite	No	242 (96.4%)	9 (3.6%)	251 (100%)
		100.0%	7.4%	69.1%
	Yes	0 (0.0%)	112 (100%)	112 (100%)
		0.0%	92.6%	30.9%

Total		242 (66.7%)	121 (33.3%)	363 (100.0%)
		100.0%	100.0%	100.0%

**Table 2:** Determinant factors of uterine rupture among females who attended maternal health services in MTUTH, MTU, 2016.

Variables	Category	SVD	Uterine rupture	Total	COR (95% CI)	AOR (95% CI)
Age	<20 yrs	39 (88.6%)	5 (11.4%)	44 (100%)	0.12	0.423
		16.1%	4.1%	12.1%	(0.040, 0.370)	(0.054, 3.281)
	20–34 yrs	183 (65.8%)	95 (34.2%)	278 (100%)	0.494	1.689
		75.6%	78.5%	76.6%	(0.255, 0.957)	(0.486, 5.868)
	>34 yrs	20 (48.8%)	21 (51.2%)	41 (100%)	Reference	Reference
		8.3%	17.4%	11.3%		

Residence	Urban	27 (81.8%)	6 (18.2%)	33 (100%)	0.671	0.854
		11.2%	5.0%	9.1%	(0.262, 1.717)	(0.195, 3.728)
	Rural	61 (48.8%)	64 (51.2%)	125 (100%)	3.168	**3.996**
		25.2%	52.9%	34.4%	(1.975, 5.082)	**(2.011, 7.940)**
	Semiurban	154 (75.1%)	51 (24.9%)	205 (100%)	Reference	Reference
		63.6%	42.1%	56.5%		

Parity	Nullipara	81 (87.1%)	12 (12.9%)	93 (100%)	0.099	0.197
		33.5%	9.9%	25.6%	(0.042, 0.231)	(0.040, 0.975)
	Multipara	143 (63.6%)	82 (36.4%)	225 (100%)	0.382	0.308
		59.1%	67.8%	62.0%	(0.199, 0.736)	(0.089, 1.064)
	Grand multiparity	18 (40.0%)	27 (60.0%)	45 (100%)	Reference	Reference
		7.4%	22.3%	12.4%		

ANC	No	60 (45.1%)	73 (54.9%)	133 (100%)	Reference	Reference
		24.8%	60.3%	36.6%		
	Yes	182 (79.1%)	48 (20.9%)	230 (100%)	0.217	**0.315**
		75.2%	39.7%	63.4%	(0.136, 0.346)	**(0.164, 0.606)**

Gestational age	Preterm	23 (82.1%)	5 (17.9%)	28 (100%)	0.114	**0.135**
		9.5%	4.1%	7.7%	(0.034, 0.382)	**(0.025, 0.725)**
	Term	208 (68.6%)	95 (31.4%)	303 (100%)	0.239	1.067
		86.0%	78.5%	83.5%	(0.111, 0.516)	(0.327, 3.490)
	Postterm	11 (34.4%)	21 (65.6%)	32 (100%)	Reference	Reference
		4.5%	17.4%	8.8%		

Preoperative hemoglobin	>7 mg/dL	234 (80.4%)	57 (19.6%)	291 (100%)	Reference	Reference
		96.7%	47.1%	80.2%		
	<7 mg/dL	8 (11.1%)	64 (88.9%)	72 (100%)	32.842	**62.517**
		3.3%	52.9%	19.8%	(14.906, 72.360)	**(22.181, 176.204)**

Sex of neonate	Female	124 (70.1%)	53 (29.9%)	177 (100%)	Reference	Reference
		51.2%	43.8%	48.8%		
	Male	118 (63.4%)	68 (36.6%)	186 (100%)	1.348	0.719
		48.8%	56.2%	51.2%	(0.869, 2.091)	(0.382, 1.352)

**Table 3:** Frequency distributions of bad outcomes of uterine rupture in MTUTH, MTU, 2016.

		SVD	Uterine rupture	Total
Maternal death	Yes	0 (0.0%)	11 (100%)	11 (100%)
		0.0%	9.1%	3.0%
	No	242 (68.8%)	110 (31.3%)	352 (100%)
		100.0%	90.9%	97.0%

Fetal loss	Yes	0 (0.0%)	107 (100%)	107 (100%)
		0.0%	88.4%	29.5%
	No	242 (94.5%)	14 (5.5%)	256 (100%)
		100.0%	11.6%	70.5%

Postoperative hemoglobin	>7 mg/dL	241 (71.3%)	97 (28.7%)	338 (100%)
		99.59%	80.17%	93.11%
	<7 mg/dL	1 (4%)	24 (96%)	25 (100%)
		0.41%	19.83%	6.89%

Obstetric complications	No	242 (98.78%)	3 (1.22%)	245 (100%)
		100%	2.48%	67.49%
	Yes	0 (0%)	118 (100%)	118 (100%)
		0%	97.52%	32.51%

Total		242	121	363
		66.7%	33.3%	100.0%
		100.0%	100.0%	100.0%

**Table 4:** Bad outcomes of uterine rupture among study participants in MTUTH, MTU, 2016.

Outcome	*β*	AI robust Std. Err.	*p* value	95% conf. interval	
				Lower	Upper
Fetal loss	**0.884**	0.029	**<0.001**	**0.827**	**0.942**
Maternal death	**0.091**	0.026	**<0.001**	**0.040 **	**0.142**
Postoperative hemoglobin	0.289	0.337	0.391	−0.949	0.371
Obstetric complication	**0.975**	0.014	**<0.001**	**0.947**	**1.000**

**Table 5:** Bad outcomes of uterine rupture among females in the catchment area by extrapolation, MTU, 2016.

Outcome	*β*	AI robust Std. Err.	*p* value	95% conf. interval	
				Lower	Upper
Fetal loss	**0.812**	0.047	**<0.001**	**0.721**	**0.904**
Maternal death	**0.035**	0.013	**0.008**	**0.009**	**0.061**
Postoperative hemoglobin	0.094	0.114	0.411	−0.318	0.130
Obstetric complication	**0.992**	0.005	**<0.001**	**0.982**	**1.000**

## Abbreviations

ANC: Antenatal care
AOR: Adjusted odds ratio
CI: Confidence interval
COR: Crude odds ratio
MTU: Mizan-Tepi University
MTUTH: Mizan-Tepi University teaching hospital
PNC: Postnatal care
SNNPR: Southern Nations, Nationalities, and Peoples' Region
SVD: Spontaneous vaginal delivery.
